# Supplementary material for: Investigation of the Interaction between Mechanosynthesized ZnS Nanoparticles and Albumin Using Fluorescence Spectroscopy
Source: Pharmaceuticals (Basel). 2023 Aug 29;16(9):1219. doi: 10.3390/ph16091219 (PMC10536685; doi:10.3390/ph16091219)
Supplement: Supplementary file 1 [file pharmaceuticals-16-01219-s001.zip › pharmaceuticals-2554023-supplementary.pdf]

Supplementary Materials

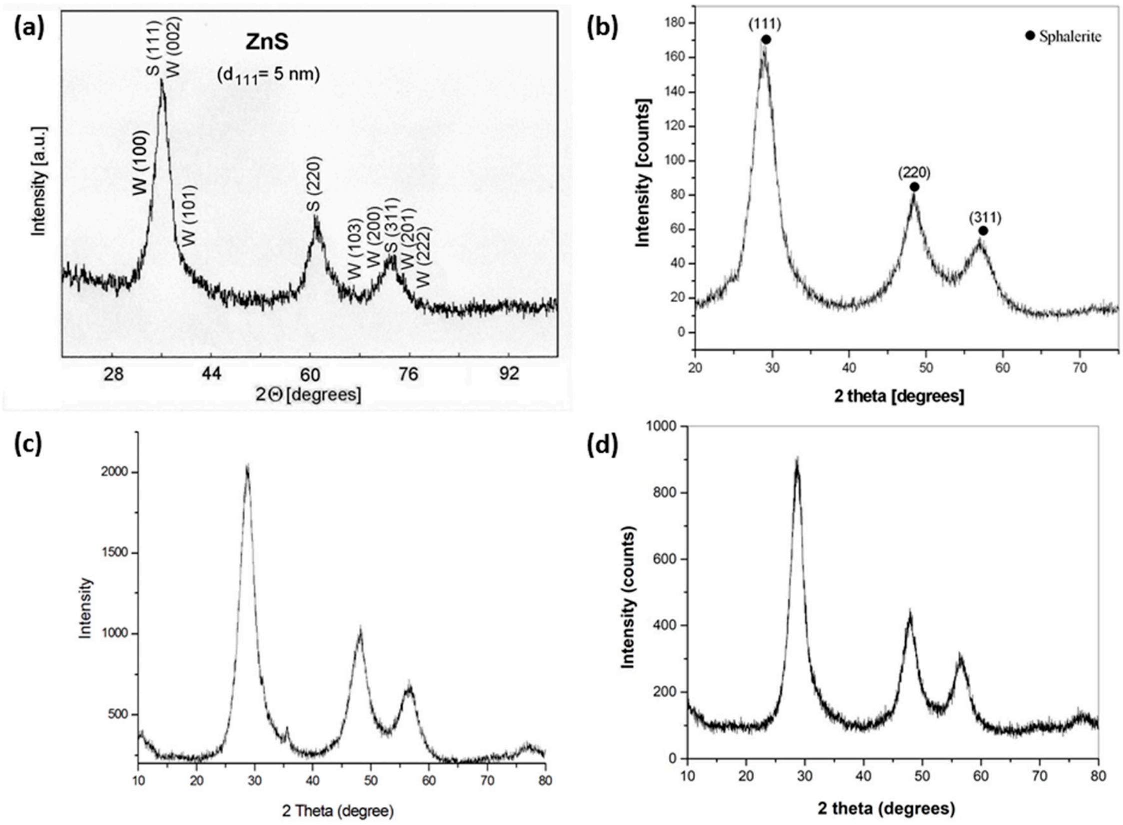

Figure S1. XRD patterns of mechanochemically synthesized ZnS nanoparticles: (**a**) the value of 5 nm was calculated by Scherrer formula. Reproduced, with permission, from Ref. [8]; (**b**) the values of 1.8 nm was calculated by surface Warren-Averbach method and 3.7 nm by volume Williamson Hall method. Reproduced, with permission, from Ref. [41]; (**c**) the value of 3.4 nm was calculated by Rietveld analysis. Reproduced, with permission, from Ref. [42]; (**d**) the value of 2.5 ± 0.5 nm was calculated by Rietveld analysis. Reproduced, with permission, from Ref. [43].

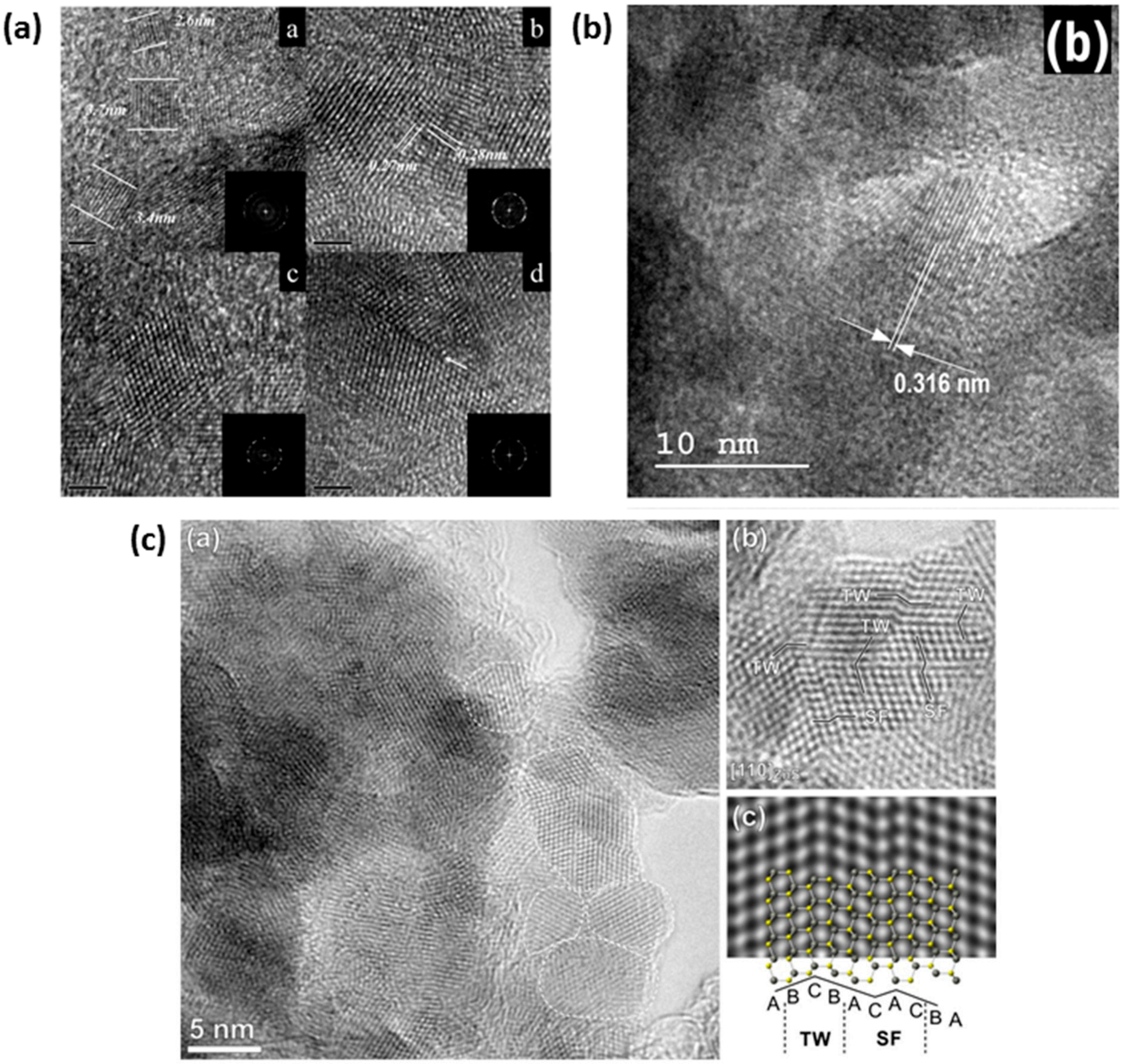

Figure S2. HRTEM patterns of mechanochemically synthesized ZnS nanoparticles. Identification of nanoparticles with sizes around: (**a**) 3 nm. Reproduced, with permission, from Ref. [41]; (**b**) 7.3 nm. Reproduced, with permission, from Ref. [42]; (**c**) 10 nm Reproduced, with permission, from Ref. [43].

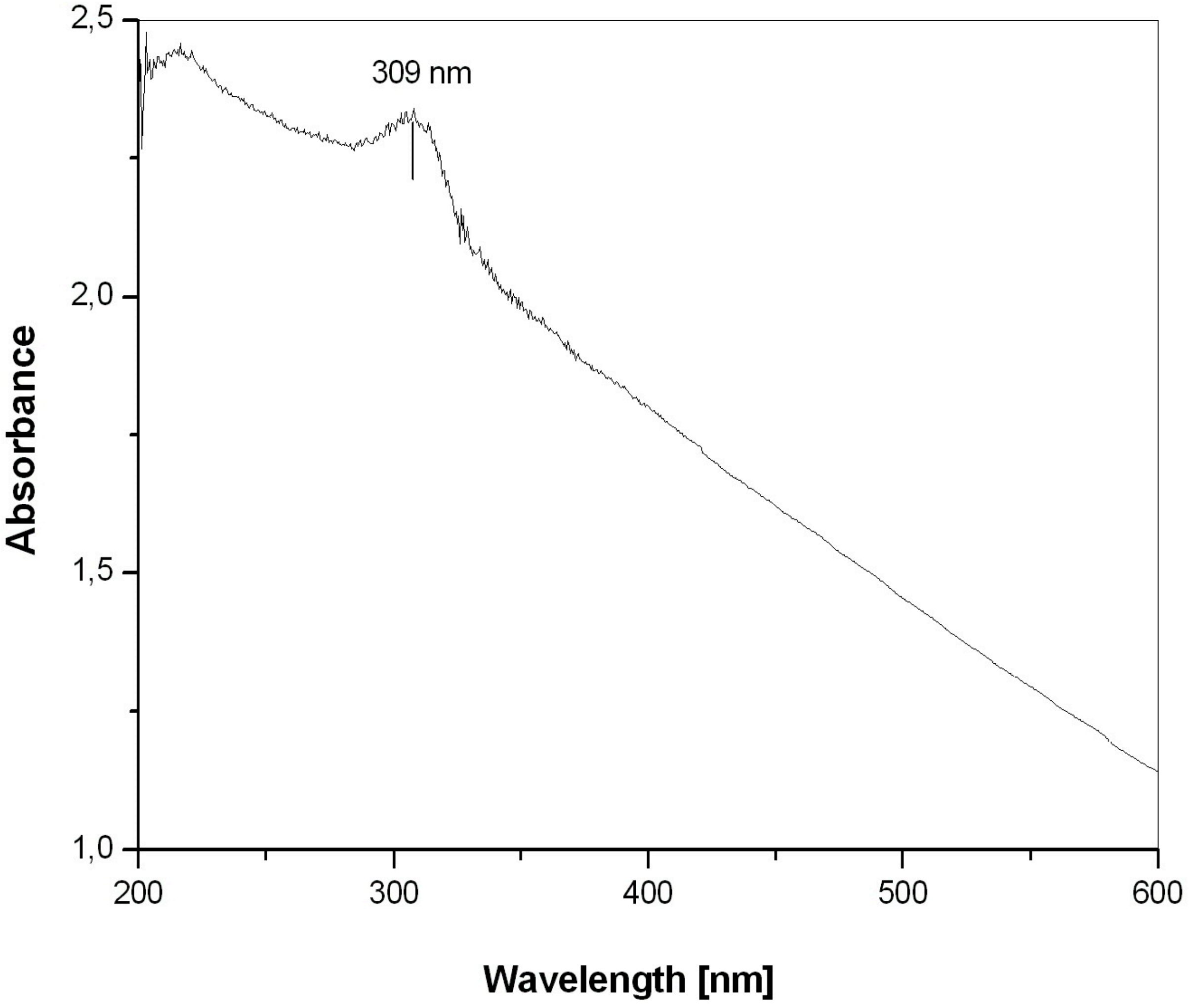

Figure S3. UV-Vis spectrum of mechanochemically synthesized ZnS nanoparticles. Reproduced, with permission, from Ref. [41].

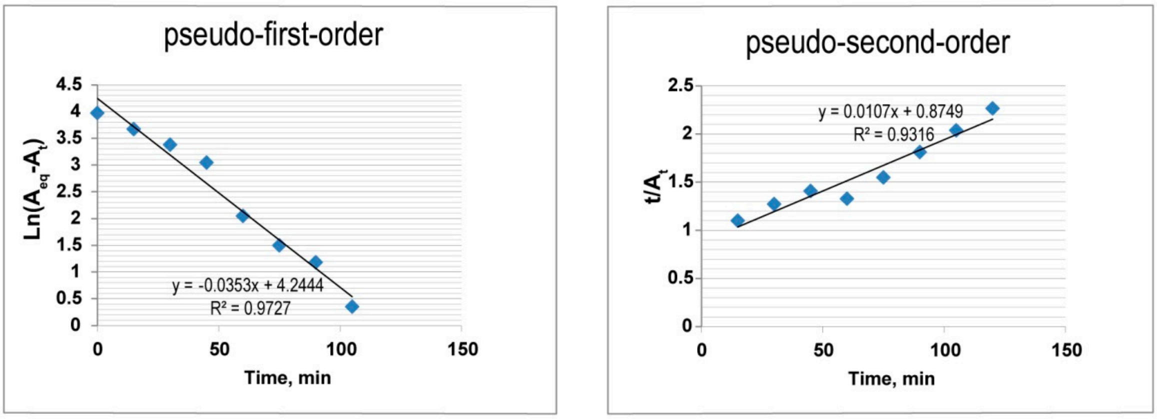

Figure S4. Linearized plots for modeling adsorption kinetics.
